# Adverse Reactions to Anticancer Drugs in the Oral Cavity

**DOI:** 10.1155/2018/1372874

**Published:** 2018-10-02

**Authors:** Olga Di Fede, Noam Yarom, Jose Bagan, Sven Otto, Stefano Fedele

**Affiliations:** ^1^Department of Surgical, Oncological and Oral Sciences, University of Palermo, Palermo, Italy; ^2^Oral Medicine Unit, Sheba Medical Center, Tel Hashomer, Israel; ^3^Maurice and Gabriela Goldschleger School of Dental Medicine, Tel Aviv University, Tel Aviv, Israel; ^4^Department of Stomatology University of Valencia, Hospital General Universitario de Valencia, Spain; ^5^Department of Oral and Maxillofacial Surgery, Ludwig Maximilian University of Munich, Munich, Germany; ^6^University College London, UCL Eastman Dental Institute London; NIHR University College London Hospitals Biomedical Research Centre, London, UK

The development, testing, and adoption into clinical practice of anticancer medications have revolutionized cancer care over the past decades. A better understanding of the biology of cancer has translated into development of novel systemic agents, as well a more effective use of older chemotherapy agents. As a consequence, cancer mortality continues to decrease.

However, greater cure and disease control rates come at a price of an increased risk of adverse effects, which often affects the mouth and related structures including the oral mucosa, salivary glands, jawbones, and cranial nerves. Oral mucositis, hyposalivation, dysgeusia, and osteonecrosis of the jaw (ONJ) are some examples of the potential adverse effects of anticancer therapies to the oral cavity, which affect an increasing number of individuals living with cancer and cancer survivors and can lead to persistent discomfort, pain, dysfunction, and a notable reduction in the quality of life. Management of these oral adverse effects can be challenging, as it typically requires a multidisciplinary approach and a close collaboration between the cancer team and oral health care providers, both in primary care and in the specialist setting.

This special issue provides a useful update of some of the most significant adverse reactions to anticancer drugs in the oral cavity, with a view to inform clinical practice and inspire further research.

Multitargeted tyrosine kinase inhibitors including sunitinib, sorafenib, axitinib, and cabozantinib are increasingly used in the cancer setting, and C. Arena et al. provide in this special issue a useful systematic review on oral mucositis associated with these agents. Similarly, K. Pimolbutr et al. report on the development of ONJ associated with antiangiogenic agents in the subset of antiresorptive-naïve patients.

Prevention of toxicity is crucial in individuals due to commence and in those who have been using antiresorptive medications, and O. Di Fede et al. discuss the main strategies to reduce the risk ONJ in this patient population.

The surgical treatment of medication-related ONJ is a relatively new field of research, as this condition has been historically managed conservatively with a focus on pain management and resolution of infection. In this special issue, R. Mauceri et al. report on the use of Er,Cr:YSGG laser and platelet-rich plasma in the surgical treatment of ONJ, whereas R. Sacco et al. provide a systematic review of the efficacy of microsurgical reconstruction of the jaws using vascularized free flap in patients with medication-related ONJ.

We hope that the readers of BioMed Research International will find this special issue interesting and informative.

